# Detailed interrogation of trypanosome cell biology via differential organelle staining and automated image analysis

**DOI:** 10.1186/1741-7007-10-1

**Published:** 2012-01-03

**Authors:** Richard J Wheeler, Keith Gull, Eva Gluenz

**Affiliations:** 1 The Sir William Dunn School of Pathology, University of Oxford, South Parks Road, Oxford, OX1 3RE, UK

## Abstract

**Background:**

Many trypanosomatid protozoa are important human or animal pathogens. The well defined morphology and precisely choreographed division of trypanosomatid cells makes morphological analysis a powerful tool for analyzing the effect of mutations, chemical insults and changes between lifecycle stages. High-throughput image analysis of micrographs has the potential to accelerate collection of quantitative morphological data. Trypanosomatid cells have two large DNA-containing organelles, the kinetoplast (mitochondrial DNA) and nucleus, which provide useful markers for morphometric analysis; however they need to be accurately identified and often lie in close proximity. This presents a technical challenge. Accurate identification and quantitation of the DNA content of these organelles is a central requirement of any automated analysis method.

**Results:**

We have developed a technique based on double staining of the DNA with a minor groove binding (4'', 6-diamidino-2-phenylindole (DAPI)) and a base pair intercalating (propidium iodide (PI) or SYBR green) fluorescent stain and color deconvolution. This allows the identification of kinetoplast and nuclear DNA in the micrograph based on whether the organelle has DNA with a more A-T or G-C rich composition. Following unambiguous identification of the kinetoplasts and nuclei the resulting images are amenable to quantitative automated analysis of kinetoplast and nucleus number and DNA content. On this foundation we have developed a demonstrative analysis tool capable of measuring kinetoplast and nucleus DNA content, size and position and cell body shape, length and width automatically.

**Conclusions:**

Our approach to DNA staining and automated quantitative analysis of trypanosomatid morphology accelerated analysis of trypanosomatid protozoa. We have validated this approach using *Leishmania mexicana*, *Crithidia fasciculata *and wild-type and mutant *Trypanosoma brucei*. Automated analysis of *T. brucei *morphology was of comparable quality to manual analysis while being faster and less susceptible to experimentalist bias. The complete data set from each cell and all analysis parameters used can be recorded ensuring repeatability and allowing complete data archiving and reanalysis.

## Background

Trypanosomatids are a family of single celled parasitic protozoa that includes several major human pathogens, *Trypanosoma brucei*, *Trypanosoma cruzi*, and *Leishmania *spp., which are responsible for a range of tropical and subtropical diseases. All trypanosomatids share similar, precisely defined, architectures; a single flagellum that emerges from an invagination of the plasma membrane at its base (the flagellar pocket), the kinetoplast (mitochondrial DNA, which can make up a large proportion of total DNA, for example, 30%, for *T. cruzi *[1,2]) found within the single mitochondrion at the base of the flagellum, a nucleus, and a subpellicular corset of microtubules that determine the cell body shape. The cell architecture undergoes regular changes during the cell cycle [3-7] as the organelles progress through a well defined series of duplicative changes [5-9]. Similar changes also occur during the various differentiation events between different life cycle stages, which have been extensively described for *T. brucei *[10-12]. Analysis of trypanosomatid morphology, and deviation from normal morphologies in mutants or following chemical insults, therefore plays an important role in understanding trypanosomatid biology. In particular tools for high-throughput, high-content, analysis of morphology are of growing importance as large sample sets, such as RNAi libraries [13] and screening of drug candidates [14], become more prevalent. These tools are, however, not well developed.

Techniques for analyzing trypanosomatid morphology are currently largely limited to manual interpretation of micrographs of cells stained with a fluorescent DNA stain and a phase contrast image. Quantitative analyses have been applied to nuclear and kinetoplast DNA [5,15] and there are many examples of quantitative analysis of cell morphological features [4,5,16-18]. These manual analyses are, however, both slow and susceptible to the bias of the experimentalist. As a result many studies are limited to 'K-N counts', where the DNA containing organelles of a cell are classified and counted manually, and quantitative analyses of DNA content or cell morphology are not made. Tools for high-throughput analysis of trypanosomatid morphology and DNA are not well developed, for example flow cytometry is of limited use in analyzing trypanosomatid DNA content as routine protocols cannot quantify the kinetoplast and nuclear DNA separately. Automated image analysis tools, such as CellProfiler [19], and their thresholding techniques [20-22] often focus on nucleus-based identification of cells, which cannot account for the two large DNA containing organelles in trypanosomatid cells. We have addressed this lack of high-throughput analysis tools by developing an automated image analysis tool optimized for use with trypanosomatid species.

Current methods for analysis of trypanosomatid cells require identification of kinetoplasts and nuclei based on number and morphology; this is susceptible to bias and may not be appropriate for some mutant phenotype analyses. An unambiguous way to identify kinetoplasts and nuclei, including in mutant or drug treated cells where organelle structure may be significantly disrupted, is desirable. Secondly, quantification of kinetoplast and nuclear DNA is currently limited by both difficulty in identifying the organelles in an unambiguous manner and then outlining the organelles for quantification from the signal in a fluorescent DNA signal. This is particularly difficult in situations where the kinetoplast and nucleus lie very close together, such as in *Leishmania mexicana *[5] and dividing *T. brucei *[4]. These issues motivated us to develop a technique which uses a minor groove binding (MGB) DNA stain (for example, 4', 6-diamidino-2-phenylindole (DAPI)) and a base pair intercalating (BPI) DNA stain (for example, propidium iodide) simultaneously then analyze the resulting staining pattern which arises due to the different affinity of the two stains to DNA with a more A-T or G-C rich composition [23-27]. We combined this with image processing by color deconvolution to separate the signal from kinetoplast and nuclear DNA to two separate images thus allowing unambiguous identification of kinetoplasts and nuclei and greatly simplifying quantitation of the kinetoplast and nuclear DNA content of a cell. In addition to developing this staining approach we used it as a basis to develop software tools to demonstrate this capability, in the form of a set of macros for the open source scientific image analysis software ImageJ [28], for high-throughput analysis of trypanosomatid morphology from micrographs.

The image analysis tools we have developed, when combined with our approach to DNA staining, have allowed us to automate the detection and analysis of cells in micrographs. Each cell is automatically analyzed to quantify kinetoplast and nuclear DNA content separately, detect and count kinetoplasts and nuclei and measure cell length and width and kinetoplast and nucleus location. This data is recorded on a cell-by-cell basis and can be matched back to the original images of the cells for manual corroboration or further analysis. Automation makes this quantitation fast, unbiased and perfectly repeatable while leaving a full record of all analysis parameters used, data collected, the data from each cell and the location of the corresponding cell in the micrographs.

## Results

### Different DNA staining dyes bind kinetoplast and nuclear DNA with different affinities

**Figure 1:**
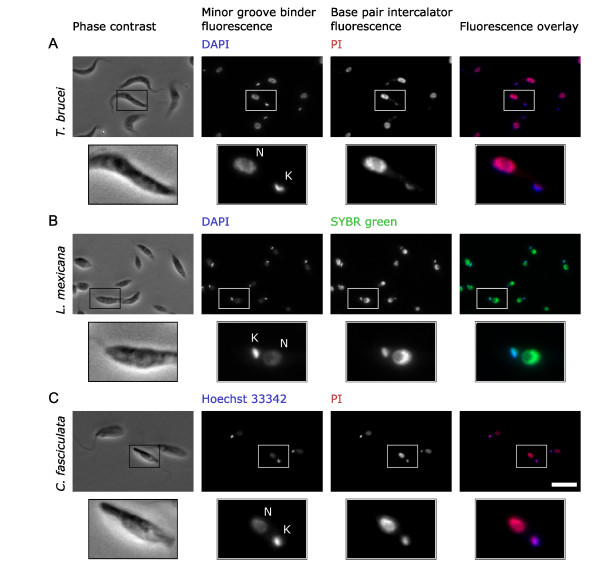
**Double labeling of kinetoplasts and nuclei with minor groove binding and base pair intercalating DNA stains**. **(A) **Micrographs of procyclic *Trypanosoma brucei *labeled with 4', 6-diamidino-2-phenylindole (DAPI) and propidium iodide (PI). **(B) **Promastigote *Leishmania mexicana *labeled with DAPI and SYBR green. **(C) **Choanomastigote *Crithidia fasciculata *labeled with Hoechst 33342 and PI. Lower panels show an enlarged view of individual cells. In each case the kinetoplast (K) appears brighter in the minor groove binding (MGB) stain image (DAPI or Hoechst 33342) than the base pair intercalating (BPI) stain image (PI or SYBR green). The reverse is true for the nucleus (N). For further examples of this effect see Additional file 1. Scale bar represents 10 μm.

Kinetoplast DNA is A-T rich in comparison to trypanosomatid nuclear DNA [29-33] and we reasoned that this would allow identification of the two organelles based on their different sequence bias by using two fluorescent DNA stains with different affinity for A-T or G-C rich DNA. MGB stains, such as DAPI [23] and Hoechst [24], have a binding preference for A-T rich sequences [23,24]. BPI stains, such as ethidium bromide [25], propidium iodide (PI) and SYBR green [26] have low sequence specificity in binding. We therefore tested staining of three trypanosomatid species, *T. brucei, C. fasciculata *and *L. mexicana*, with two DNA stains simultaneously; one BPI stain and one MGB stain. The combinations of stains tested were DAPI with SYBR green, DAPI with PI and Hoechst 33342 with PI (Figure 1 and Additional file 1). For all species and stain combinations the kinetoplast appeared brighter, that is, was labeled more strongly, with the MGB stain than with the BPI stain and the nucleus appeared brighter with the BPI stain than with the MGB stain. This difference in staining with BPI and MGB DNA stains provided us a means by which to identify kinetoplasts and nuclei based purely on the sequence bias of their DNA.

### Color deconvolution can separate signal from kinetoplast and nuclear DNA based on an MGB and BPI stain

Both DNA stains show the same structures, but the intensity of signal depends on the sequence bias of the DNA in the organelle and the stain used. The BPI and MGB stain images can be thought of as two color components where the different binding properties of the BPI and MGB stain give double-stained kinetoplasts and nuclei a different two dimensional color (that is, two color channels, analogous to red and blue from an RGB color image), as shown in the color overlays in Figure 1 and Additional file 1.

Color deconvolution acts to separate features in images based on their color and has been used with micrographs for quantitative histochemistry [34] and analysis of multicolor fluorescence *in situ *hybridization for color compensation [35,36]. We applied this technique to separate the contribution of kinetoplast and nuclear DNA to the MGB and BPI stain images allowing us to generate two separate images, one containing only kinetoplasts and the other containing only nuclei. This simplifies automated and manual analysis of the micrographs as it eliminates the need to identify kinetoplasts and nuclei based on their morphology or position within the cell.

In order to perform accurate color deconvolution, two image preparation steps were required. Firstly, because color deconvolution requires precise alignment of the two florescence images, chromatic aberration must be measured and corrected [37]. Secondly color deconvolution requires reference values that represent the color of a typical kinetoplast and nucleus, in this case these reference values are the average intensity of kinetoplast and nuclear DNA in the MGB and BPI images (see Methods). Our set of tools, written for use in ImageJ [28], for the analysis of trypanosomatid cells and their DNA by color deconvolution can automate these steps.

### Preparing for color deconvolution: adjusting for chromatic aberration

Chromatic aberration in a fluorescence microscope results in slightly different magnifications for images captured at different emission wavelengths [38]. We showed the impact of this with DAPI-stained and PI-stained procyclic *T. brucei *where color deconvolution without correcting for chromatic aberration gave strong fringes around the kinetoplast and nucleus while color deconvolution following correction of chromatic aberration did not (Additional file 2). Lateral chromatic aberration can be corrected with a linear scaling of one image, but the precise strength of the effect must first be measured. Our chromatic aberration tool used the kinetoplasts and nuclei present within the sample to calculate the strength of aberration, eliminating the need for a separate calibration sample as used by some other tools [38]. Following measurement of chromatic aberration (Additional file 3, part A) the aberration was corrected by scaling of the BPI image (Additional file 3, part B).

### Preparing for color deconvolution: calculation of reference values

**Figure 2:**
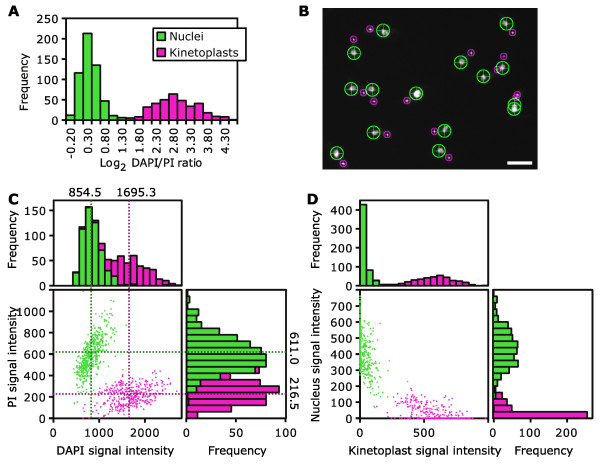
**Calculation of reference values for and application of color deconvolution**. **(A) **Histogram of log_2 _4', 6-diamidino-2-phenylindole (DAPI)/propidium iodide (PI) signal ratios from 897 points (single pixels) automatically identified from 3 fields of view of procyclic *Trypanosoma brucei *stained with DAPI and PI. Each point samples a single kinetoplast or nucleus except in rare cases (<5%) where multiple points were identified per organelle. Points were automatically assigned to either a high log_2 _ratio (536 points) or a low log_2 _ratio (361 points) group. **(B) **An example region of one of the source DAPI fluorescence micrographs used to generate the data in (A). Each point analyzed is marked with a ring color coded to indicate whether it fell into the high (magenta) or low (green) log_2 _ratio group. Scale bar represents 10 μm. **(C) **DAPI and PI values of the 897 example points. Points were identified as nuclei or kinetoplasts as described in (A) and (B). The average DAPI and PI signal for kinetoplasts and nuclei, which are the reference values for color deconvolution, are indicated. **(D) **The kinetoplast and nuclear signal values of the same 897 points following color deconvolution. Color deconvolution acts, using the reference values, to maximize the separation of signal from kinetoplasts and nuclei.

In order to perform color deconvolution two reference values for the intensity of signal from kinetoplast and nuclear DNA in the BPI and MGB fluorescence images are required, representing the two-dimensional color of kinetoplasts and nuclei when observed with these stains. We tested the performance of our color deconvolution tool for measuring these reference values automatically on a test sample of 3 fields of view (236 cells) of procyclic *T. brucei *stained with DAPI (MGB) and PI (BPI). Reference values were determined by automatically finding bright points, that is, kinetoplasts and nuclei, in the fluorescence images and measuring the log_2 _ratio of signal intensity in the MGB stain to the BPI stain image for each point. In all, 897 points were analyzed. A histogram of the log_2 _intensity ratios showed a clear bimodal distribution and the color deconvolution tool used one-dimensional *k*-means clustering to assign automatically the points in this histogram to two groups (Figure 2A). From the DNA sequence bias of the organelles and the staining properties of the MGB and BPI stains we expected the high and low log_2 _intensity ratio clusters to correspond to kinetoplasts and nuclei, respectively.

To confirm this expectation we mapped the assignment of each point back to the source images and manually assessed whether they had been correctly assigned (Figure 2B). Of the 897 points analyzed only 2 were assigned incorrectly. Finally the average signal in the BPI and MGB stain images was calculated for all points classified as kinetoplasts and then nuclei (Figure 2C). The resulting four values are the reference values for color deconvolution (Figure 2D).

Small variations in the production and staining of samples made the reference values vary from sample-to-sample. When making a quantitative comparison between samples this would be an issue as sets of color-deconvolved images can only be compared quantitatively when identical color deconvolution reference values have been used [34]. We therefore optimized two methods of staining samples (see Methods). The first (method 1) was optimized for photostability of the DNA stains. The second (method 2) was optimized for minimum sample-to-sample variation to allow quantitative sample comparison. To demonstrate typical variation, when using method 2, in staining we captured four sets of images of DAPI-stained and PI-stained procyclic *T. brucei *from four samples prepared in parallel on a single slide. Using the color deconvolution tool we recorded the DAPI and PI intensities of 1,300 kinetoplasts and nuclei from each image set (Additional file 4). Variation between samples was low; the measured ratio of DAPI to PI signal was 0.62 ± 0.08 and 2.48 ± 0.24 (mean ± standard deviation of the four samples) for nuclei and kinetoplasts, respectively. This variation is small enough to allow us to use one sample to calculate kinetoplast and nucleus reference values, and then apply these values to perform color deconvolution on images from a second sample for quantitative comparison.

### Performing color deconvolution

**Figure 3:**
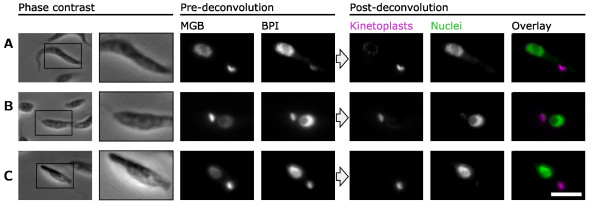
**Separation of kinetoplast and nuclear signal by color deconvolution**. Micrographs of cells, as shown in Figure 1, before and after color deconvolution. **(A) **Procyclic *Trypanosoma brucei *labeled with 4', 6-diamidino-2-phenylindole (DAPI) (minor groove binding (MGB)) and propidium iodide (PI) (base pair intercalating (BPI)). **(B) **Promastigote *Leishmania mexicana *labeled with DAPI (MGB) and SYBR green (BPI). **(C) **Choanomastigote *Crithidia fasciculata *labeled with Hoechst 33342 (MGB) and PI (BPI). Scale bar represents 5 μm.

**Figure 4:**
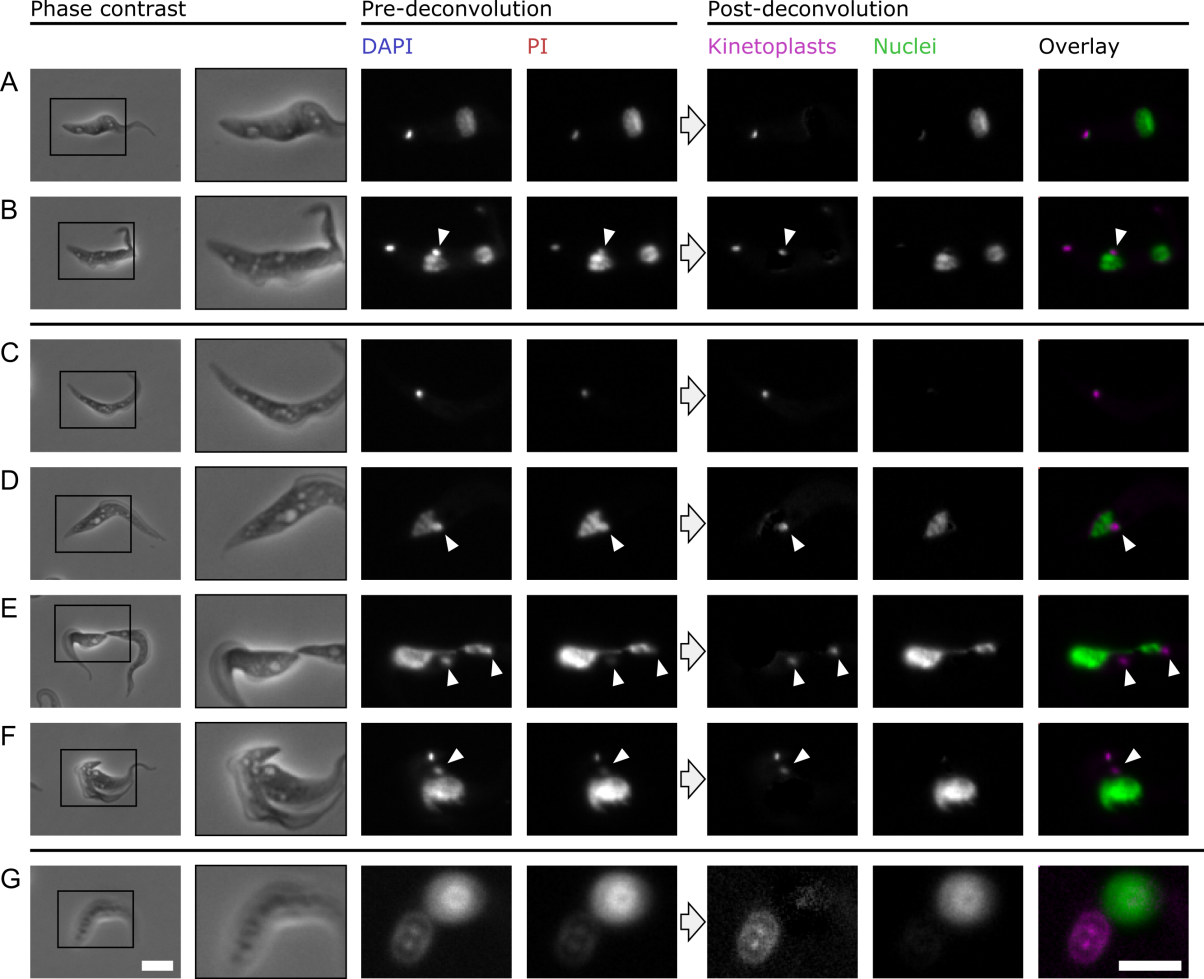
**Color deconvolution can separate kinetoplast and nuclear DNA signal in *Trypanosoma brucei *even when the organelles are closely apposed or abnormal in structure**. 4', 6-Diamidino-2-phenylindole (DAPI) and propidium iodide (PI) fluorescence and color-deconvolved images of procyclic *T. brucei *48 h after induction of expression of sister chromatid cohesion protein 1 (SCC1)-mutAB. **(A) **1K1N cell. **(B) **2K2N cell. Signal from the closely apposed anterior kinetoplast and posterior nucleus (arrowhead) can be separated. **(C) **Zoid (1K0N) cell illustrating a lack of nuclear DNA. **(D) **Cell with zoid-like DAPI staining which possess a fragment of nucleus next to the kinetoplast (arrowhead). **(E,F) **Cells undergoing aberrant cytokinesis, having failed to undergo mitosis, with a severely distorted nuclear structure. Color deconvolution can unambiguously identify kinetoplasts (arrowheads) among nuclear DNA fragments. **(G) **An out of focus 2K1N cell; kinetoplast and nucleus signal can still be accurately separated by color deconvolution despite being out of focus. Scale bar represents 5 μm.

Color deconvolution takes the signal from kinetoplasts and nuclei in the BPI and MGB stain images and separates it according to the reference values (Figure 2D), reassigning the fluorescent signal to two new images, one which contains only the kinetoplasts and the other which contains only the nuclei. We demonstrated this with *T. brucei, L. mexicana *and *C. fasciculata *(Figure 3) and mutant *T. brucei *cells (Figure 4). Importantly, this transformation preserves the quantitative nature of staining DNA with a fluorescent molecule [15,34], therefore quantity of DNA in the kinetoplasts and nuclei can be measured from the deconvolved images. Furthermore when staining and color deconvolution is performed appropriately, that is, using staining method 2 (see Methods) and using identical values for color deconvolution, multiple samples can also be compared quantitatively.

### Color deconvolution performs well on aberrant morphologies

When using double DNA staining and color deconvolution the morphology of the organelle and its location within the cell do not need to be taken into consideration to determine whether it is a kinetoplast or nucleus. This allows unbiased analysis of samples where the cell, kinetoplast or nucleus morphology is significantly altered.

We demonstrated this using a procyclic *T. brucei *cell line expressing a non-cleavable sister chromatid cohesion protein 1 (SCC1) allele (SCC1-mutAB [39]). SCC1-mutAB cannot be cleaved by separase so it has a dominant negative effect on the onset of anaphase. In procyclic *T. brucei *this results in cells with abnormal morphologies, typically anucleate cytoplasts with one kinetoplast ('zoids') and cells with large nuclei [39]. SCC1-mutAB expression was induced for 48 h then cells were stained with DAPI and PI and the micrographs were analyzed by color deconvolution. Color deconvolution was able to separate signal from kinetoplasts and nuclei for all cells, including cells with approximately wild-type morphology (Figure 4A,B), zoids (Figure 4C), cells with disrupted nuclear structure (Figure 4D-F) and out-of-focus cells (Figure 4G).

### Kinetoplast and nuclear DNA can be quantified from color-deconvolved images

Color deconvolution simplifies the separate quantitation of nuclear and kinetoplast DNA content. Analysis of kinetoplast and nuclear DNA content of a cell from a micrograph first requires identification of the organelles and segmentation of them from the image, the total signal intensity for each organelle can then be recorded [15]. With color-deconvolved images the fluorescence signal from DNA has been split to two separate images; there is no longer a requirement to manually segment kinetoplasts and nuclei from the image, instead the kinetoplast and nucleus DNA content can be measured from the total signal in the kinetoplast or nucleus image within the boundary of the cell.

**Figure 5:**
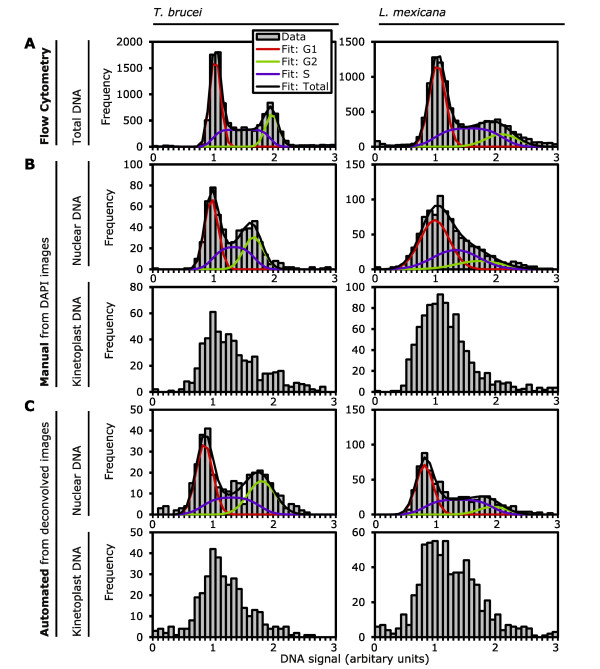
**Comparison of three methods for quantifying trypanosomatid DNA**. The DNA content of exponentially growing procyclic *Trypanosoma brucei *and promastigote *Leishmania mexicana *was analyzed by three different methods. **(A) **Histograms of total DNA content of cells as measured by flow cytometry of propidium iodide (PI)-stained and RNaseA-treated cells (n = 10,000). Kinetoplast and nuclear DNA cannot be quantified separately by this method. **(B) **Histograms of nuclear and kinetoplast DNA content cells as measured by quantification of signal from manually outlined kinetoplasts and nuclei in 4', 6-diamidino-2-phenylindole (DAPI) fluorescence images of RNaseA-treated cells (*T. brucei *n = 552, *L. mexicana *n = 932). **(C) **Histograms of nuclear and kinetoplast DNA content as measured by quantification of signal within the cell boundary from the kinetoplast and nuclear images generated from color deconvolution of micrographs of cells stained with DAPI and PI (*T. brucei*, n = 386) or SYBR green (*L. mexicana *n = 790). Flow cytometry and nuclear DNA histograms were further analyzed by fitting of a model (black line) of the G1 (red), S (purple) and G2 (green) phases of the cell cycle, the results of which are shown in Table 1.

We validated this color deconvolution approach to DNA quantification by comparing it with two pre-existing methods; flow cytometry of PI-stained cells and quantification of DAPI signal from manually segmented micrographs of DAPI-stained cells. We analyzed both exponentially growing procyclic *T. brucei *and promastigote *L*. *mexicana *with each method. Histograms of total DNA content as measured by flow cytometry (Figure 5A) and kinetoplast and nuclear DNA content as measured from manually segmented DAPI images (Figure 5B) and color-deconvolved images (Figure 5C) indicated all three methods were effective. Flow cytometry gave the expected bimodal distribution of DNA content (corresponding to cells in G1 and G2) for an exponentially growing population of cells, cells with an intermediate DNA content are in S phase (Figure 5A). The bimodal distribution was less well resolved for *L. mexicana *consistent with a long S phase, short G2 and fast cytokinesis relative to *T. brucei *[5]. Unlike flow cytometry the two micrograph-based techniques, quantification from manually segmented DAPI images and quantification from color-deconvolved images, can quantify kinetoplast and nuclear DNA separately (Figure 5B,C). Quantification of nuclear DNA from manually segmented DNA images gave a clear bimodal distribution for *T. brucei *but not *L. mexicana *where overlap of the kinetoplast and nuclei prevented accurate segmentation. Quantification of nuclear DNA from the color-deconvolved images did not suffer from this limitation and was able to resolve the expected bimodal distribution for both species. Neither technique could resolve the expected bimodal distribution of kinetoplast DNA, however the skewed distribution is consistent with underlying G1/S/G2 phase changes in DNA content.

The accuracy and precision of each method was assessed numerically by fitting a model of DNA content change through the cell cycle to the histograms of nuclear DNA content and flow cytometry data, the results of which are shown in Table 1. The ratio of the mean of the peak due to cells in G2 with those in G1 gave a measure of accuracy, with an expected value of 2. The standard deviation of DNA signal in the G1 and G2 peak gave a measure of precision. All three methods had similar accuracy with a G2/G1 ratio within 0.4 of the expected value of 2. The precision of all three methods was also similar with a standard deviation of around 0.2, flow cytometry was most precise and manual quantification from micrographs was least precise. Both microscopy-based techniques were, however, able to quantify kinetoplast and nuclear DNA separately which flow cytometry cannot.

**Table 1:** Accuracy and precision of DNA quantitation by different methods

Species	Data source	Ratio of means	Standard deviation	
			
		(post-S/pre-S)	Pre-S	Post-S
*Trypanosoma brucei*	Flow cytometry	1.83	0.08	0.10
*T. brucei*	Manual (nucleus)	1.62	0.11	0.16
*T. brucei*	Auto (nucleus)	1.90	0.14	0.24
*Leishmania mexicana*	Flow cytometry	2.07	0.13	0.26
*L. mexicana*	Manual (nucleus)	1.85	0.26	0.41
*L. mexicana*	Auto (nucleus)	2.38	0.17	0.29

Histograms of total DNA content as determined by flow cytometry, nuclear DNA content as measured manually from 4', 6-diamidino-2-phenylindole (DAPI) images (manual) and nuclear DNA content as measured automatically from color-deconvolved images (auto) were fitted to a model of pre-S-phase, post-S-phase and during S-phase changes in DNA content through the cell cycle (Figure 5). The fitting parameters are summarized this table, all values are normalized to the measured pre-S-phase DNA for that data source. The standard deviation required to match the experimental error in the pre-/post-S-phase peaks is a measure of precision. The ratio of the mean of the post-S-phase to the pre-S-phase peak gives a measure of accuracy and is expected to be 2.

### Moving beyond K and N counts to quantitative morphometric analysis

Our staining and color-deconvolution methods have greatly simplified two important aspects of automated analysis of trypanosomatid cells: identification of kinetoplasts and nuclei and separate quantification of kinetoplast and nuclear DNA. We combined this with analysis of the cell body shape to allow extraction of measures of kinetoplast and nucleus DNA content, size and location and cell body length and width. The result was a tool for ImageJ for high-content, high-throughput analysis of trypanosomatids that extracts data from three images (the phase contrast image and the color-deconvolved images of the kinetoplasts and nuclei) of fields of view of trypanosomatid cells. Our automated analysis tool takes the following approach to each analysis.

**Figure 6:**
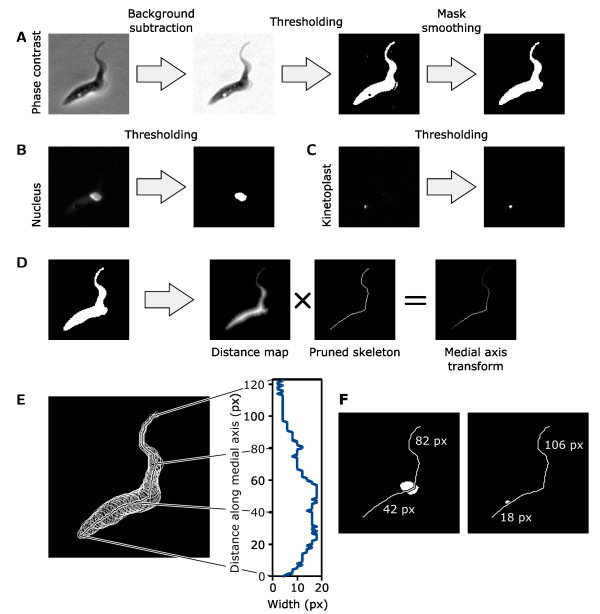
**Methods for analysis of trypanosomatid morphology, facilitated by use of color deconvolution**. **(A) **Overview of the image analysis used to identify cells from the phase contrast image. A rolling ball filter was used to remove the phase contrast halo and the resulting image was thresholded then smoothed to generate a cell mask. **(B,C) **Nuclei and kinetoplasts were identified by thresholding the nucleus and kinetoplast images from color deconvolution. **(D) **Analysis of cell morphology used the medial axis transform (MAT) of the cell mask, generated by multiplication of the Euclidian distance map and pruned skeleton of the cell mask. **(E) **The MAT is a line that follows the midline of the cell mask. Values along the MAT encode the half width of the cell at each point; the original mask shape can be reconstructed by placing a circle of the appropriate radius at every point along the MAT (left). Plotting the values of the MAT provides the cell profile shape (right). **(F) **The distance of the kinetoplast and nucleus from the two ends of the cell were measured along the MAT. The distance from the centroid of the nucleus/kinetoplast to the MAT gave the distance from the cell midline.

The cells are first identified based on the phase contrast image. A rolling ball background subtraction filter [40] is used first to reduce the strength of the bright 'phase halo' effect in the phase contrast image by smoothing large scale intensity variations but preserving fine detail. Next, a threshold is applied and the resulting mask is smoothed and small particles are removed to gives an outline of the cells (Figure 6A). Two analyses are performed on the color deconvolution images of kinetoplasts and nuclei; firstly the sum pixel value within each cell outline in each image is measured to give the total kinetoplast and nuclear DNA content of each cell. Secondly a threshold is applied to the color-deconvolved images of the kinetoplasts and nuclei to generate kinetoplast and nuclei masks (Figure 6B,C). Each kinetoplast and nucleus mask is assigned to a particular cell if any part lies within the cell's mask; this gives an automated count of the kinetoplast and nucleus number. The sum pixel value within each kinetoplast and nucleus mask is also measured to give a measure of DNA content of each organelle individually.

The medial axis transform (MAT), a mathematical descriptor of object shape, [41] is used to analyze the shape of the cell, a technique that has been used in several automated cell morphology analyses [42,43]. The MAT is produced by multiplication of the Euclidian distance map of the cell mask (where each pixel within the cell mask is given a value equal to the distance to the nearest edge of the cell mask) with its skeleton (a single pixel wide line that runs down the midline of the mask) (Figure 6D). In the case of long thin objects such as trypanosomatid cells the MAT is generally a single line that runs along the long axis of the cell. Our approach can only analyze the morphology of cells where this is the case, approximately 90% of *T*. *brucei *cells met this criterion. The MAT encodes the width of the cell for every point along its length and is a powerful tool as it can be used to make a near perfect reconstruction of the cell shape that is made by placing a circle at every point along the MAT with a radius equal to the MAT value at that point (Figure 6E). The MAT can therefore be used to give a measure of cell length, maximum width and profile shape (Figure 6E). By reference to the MAT the position of kinetoplasts and nuclei within the cell are automatically determined. The distance from an organelle to the posterior and anterior end of the cell is given by the distance of the organelle's centroid along the MAT (Figure 6F) and the distance of an organelle from the midline of the cell is given by the minimum distance to the MAT.

### Data collection and workflow

Our automated cell analysis tool is a method for extracting data from micrographs; it records all morphological and DNA content data from every object recognized from the phase contrast image. The data from each object is recorded with a link back to its location in the original micrographs and the complete data set is made available for analysis by the user and the settings used for image analysis can be recorded (Additional file 5).

Every analysis of the automatically collected data set must start by applying exclusion criteria to remove data from cells that either should not be analyzed or cannot be accurately analyzed. Cells that are touching the image edge, based on their dimensions and location within the image, should be excluded as it unclear if the whole cell is visible. Objects with no kinetoplast or nucleus that are not cells but are in fact debris or artifacts from thresholding the phase contrast images should also be excluded. Finally, objects that are in fact two cells lying in contact with each other should also be excluded. It is more difficult to exclude these in unbiased manner, especially for mutants that have altered morphology. For analyses of non-mutant *T. brucei *we excluded any cell with a branched skeleton that, with confirmation via visual examination of the images, was effective at removing cells that were touching another cell or were partially out of focus. For our analyses of mutants we did not use any exclusion criteria as it may have filtered out cells where morphology deviated significantly from the wild-type, although cells could still be excluded from the analysis on an individual basis by manual cross reference of the data with the original micrographs.

### Examples of automated analysis: changes in morphology during the *T. brucei *wild-type cell cycle

To evaluate the automated analysis of kinetoplast and nucleus DNA content and number, cell length and kinetoplast position we prepared a sample of logarithmically growing procyclic *T. brucei*, which was analyzed as described above. A total of 952 cells (from 30 images) were analyzed. Cells with a branched skeleton were not analyzed.

Cells were automatically classified by their kinetoplast and nucleus number: 70.6% 1K1N, 15.3% 2K1N, 4.8% 2K2N and 9.3% other. A manual count of kinetoplast and nucleus number of 500 cells from the same sample gave a similar result: 78.8% 1K1N, 15.0% 2K1N, 5.2% 2K2N and 0.1% other, and both are similar to previously published data [6]. The automated analysis classified more cells as 'other' than the manual analysis, which reflects errors made in classification. Some of these could have been corrected by a manual crosscheck of the original images (all 'others' can be easily identified for manual reanalysis) while others were simply too unclear to classify. The automated analysis tool does not have a propensity to assign unclear cells into the categories 1K1N, 2K1N or 2K2N, which are expected for wild-type *T*. *brucei*.

**Figure 7:**
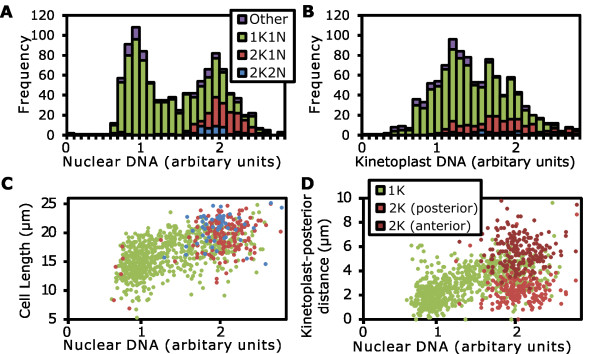
**Automated analysis of procyclic *Trypanosoma brucei *DNA content, shape and organelle positioning**. Logarithmically growing procyclic *T. brucei *were analyzed using 4', 6-diamidino-2-phenylindole (DAPI) and SYBR green staining followed by color deconvolution and automated image analysis of DNA content and morphology. **(A) **Histogram of the nuclear DNA content of cells broken down by kinetoplast and nucleus number; 1K1N (green), 2K1N (red), 2K2N (blue) and other (purple). **(B) **Histogram of the kinetoplast DNA content of cells broken down by kinetoplast and nucleus number as in (A). **(C) **Scatter plot of cell length against nuclear DNA content for 1K1N, 2K1N and 2K2N cells, color coded as in (A). **(D) **Scatter plot of kinetoplast position against nuclear DNA content. Points are shown in green for cells with a single kinetoplast and red for cells with two kinetoplasts. The more posterior kinetoplast in 2K cells is shown in the lighter shade of red.

A histogram of nuclear DNA content of cells demonstrated 2K1N and 2K2N cells had duplicated nuclear DNA content (Figure 7A). A similar histogram of kinetoplast DNA content did not give a clear bimodal distribution, but 2K1N and 2K2N cells are positioned towards the top end of the distribution (Figure 7B). Both these results are as would be expected from existing data on the *T. brucei *cell cycle [6]. The length the cell body was automatically measured for each cell using the MAT. The average length of cells was 16.3 ± 3.1 μm (1K1N), 19.6 ± 3.0 μm (2K1N) and 21.0 ± 3.3 μm (2K2N) (mean ± standard deviation). For comparison, the average length of 50 1K1N cells manually measured from the same sample was 16.8 ± 3.0 μm. These length changes through the cell cycle are consistent with previous descriptions of procyclic *T. brucei *[18]. The nature of our data set allows us to take this analysis further; plotting the cell length against nuclear DNA content shows that during nuclear G1 cells grow in length from 14 to around 20 μm and that cell length remains approximately constant at 19 to 21 μm during S phase and G2 (Figure 7C).

The morphology analysis tool also records the distance of a kinetoplast or nucleus from both ends of the cell. As the kinetoplasts always lie closer to the posterior end of the cell the minimum distance from a kinetoplast to one end of the cell is the kinetoplast-posterior distance. Plotting the kinetoplast-posterior distance against nuclear DNA content reveals the movement of the kinetoplast during the cell cycle (Figure 7D). During G1 the single kinetoplast lies at approximately 2 μm from the posterior. This distance increases during nuclear S phase reaching 4 μm at which point kinetoplast division occurs and the two daughter kinetoplasts separate to 2 μm and 6 μm from the cell posterior, respectively. This quantitative description of kinetoplast position during division is consistent with existing descriptions of basal body movement during the cell cycle [18] and division of the kinetoplast [44].

### Examples of automated analysis: using color deconvolution to quantify DNA in *T. brucei *expressing SCC1-mutAB

We demonstrated the capabilities for comparative analysis of nuclear and kinetoplast DNA by this method using a procyclic *T. brucei *expressing a non-cleavable SCC1 allele (SCC1-mutAB [39]). Following induction of SCC1-mutAB and the resulting failure of mitosis and aberrant cytokinesis it may be possible for further rounds of nuclear DNA synthesis to occur [39], although this has not previously been demonstrated quantitatively. We therefore tested this hypothesis with our automated analysis tool. Uninduced and 48 h induced samples were prepared in parallel on the same slide and 3,201 cells (uninduced sample, from 90 images) or 2,014 cells (48 h induced sample, from 120 images) were analyzed. Cells with a branched skeleton were not excluded to ensure no cells with unusual morphologies were inadvertently removed from the analysis.

**Figure 8:**
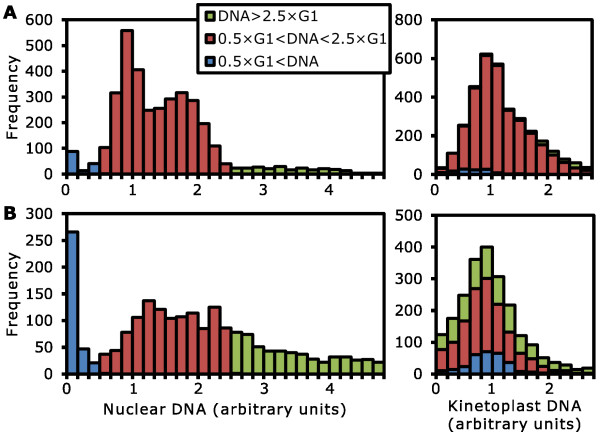
**Quantitation of nuclear DNA loss and increase in a procyclic *Trypanosoma brucei *cell line following induction of expression of sister chromatid cohesion protein 1 (SCC1)-mutAB**. Procyclic *T. brucei *before and 48 h after induction of expression of SCC1-mutAB were analyzed using 4', 6-diamidino-2-phenylindole (DAPI) and propidium iodide (PI) staining followed by color deconvolution and automated image analysis of DNA content and morphology. **(A) **Histograms of nuclear (left) and kinetoplast (right) DNA content of the uninduced cell line. Cells were categorized as normal (red), abnormally low (blue) or abnormally high (green) nuclear DNA content. **(B) **Histograms of nuclear (left) and kinetoplast (right) DNA content, at the same scale as (A), following induction for 48 h. Cells were categorized as normal, low or high nuclear DNA content based on the uninduced cell line. The proportion of cells with abnormally low or high nuclear DNA content has increased. Cells with abnormally high or low nuclear DNA content show no skew towards high or low kinetoplast DNA content.

Histograms of nuclear DNA content for the uninduced and 48 h induced samples revealed the effect of SCC1-mutAB (Figure 8). The nuclear DNA content of the uninduced cells had a distribution (Figure 8A) similar to that seen for the wild-type (Figures 5C and 7A). From this we estimated the G1 quantity of DNA and then subcategorized the cells to three groups by nuclear DNA content; cells with normal (0.5 to 2.5 × G1 DNA content), low (less than 0.5 × G1 DNA content) and high (greater than 2.5 × G1 DNA content). The distribution of nuclear DNA content for the 48 h induced cells is very different to the uninduced sample (Figure 8B) and, using the value of G1 DNA content from the uninduced sample as a reference we subcategorized the cells in the 48 h induced sample into low, normal and high nuclear DNA content. Cells with abnormally low nuclear DNA content increased from 4.1% to 16.5% and cells with abnormally high nuclear DNA content increased from 6.8% to 27.3% following induction of SCC1-mutAB for 48 h. In contrast there was little change in the distribution of kinetoplast DNA content in the induced sample (Figure 8B) in comparison to the uninduced sample (Figure 8A) and the wild-type (Figures 5C and 7A). Cells with abnormally high or low nuclear DNA content show no bias to higher or lower than normal kinetoplast DNA in either the induced or uninduced sample (Figure 8).

Taken together, these histograms show that the induction of SCC1-mutAB expression causes a disruption of segregation of nuclear DNA while kinetoplast division is unaffected. The number of cells with very low nuclear DNA content and approximately normal kinetoplast DNA content (zoids) increases, consistent with initial characterization of this mutant [39] and there is also an increase in the number of cells with abnormally high nuclear DNA content but normal kinetoplast DNA content, these may be cells which have progressed through aberrant cytokinesis, producing a zoid daughter cell, then re-entered the cell cycle and progressed through S phase increasing the nuclear DNA content to abnormally high levels. The proportion of zoids we inferred after 48 h induction of SCC1-mutAB (16.5%) is smaller than previously described (38%) [39]. We suggest that this discrepancy occurs due to overestimation of the number of zoids in the previous study. The automated analysis suggests that many cells that look like they contain only a single kinetoplast also contain a significant quantity of nuclear DNA in a partial nucleus remnant (Figure 4D,E).

## Discussion

Our approach for double DNA staining and color deconvolution unambiguously identifies the kinetoplast and nucleus in micrographs of trypanosomatids. This allows the analysis of complex trypanosomatid phenotypes without the risk of bias; our approach avoids any manual identification of kinetoplasts and nuclei that would require a qualitative judgment based on morphology, quality of staining and location of organelle within the cell. This unambiguity allowed us to draw conclusions about kinetoplast and nucleus number and structure in an example cell line, the SCC1-mutAB mutant, which would not be possible from a single DAPI micrograph. For example identification of closely apposed kinetoplasts and nuclei (Figure 4B,E) and nuclear fragments (Figure 4E,F) became trivial following color deconvolution. Analysis of SCC1-mutAB showed that cells that resembled zoids often included a fragment of nucleus adjacent to the kinetoplast, indicating that in the absence of mitosis cytokinesis can still result in partial segregation of nuclear DNA. Notably, our technique also works on out-of-focus kinetoplasts and nuclei (Figure 4G), which enables accurate identification of organelles even in monstrous cells where accurate focusing on all cellular substructures may not be possible.

Separation of fluorescent signal from kinetoplasts and nuclei to two separate images by color deconvolution provides new opportunities for quantitative analysis of the DNA content of trypanosomatids. Automated measurement of DNA from these images was faster and less susceptible to experimentalist bias than quantification from manually segmented DAPI images and produced data of greater precision, approaching the precision of flow cytometry, while quantifying kinetoplast and nuclear DNA separately (Figure 5). This was particularly evident for measurement of nuclear DNA content of *L. mexicana*: manual segmentation of the images could not account for the overlap of kinetoplast and nucleus signal due to the close proximity of these organelles [5] while automated analysis using color deconvolution could, improving precision.

We have also shown that automated quantification of the DNA content of kinetoplasts and nuclei from color-deconvolved images can be used for quantitative comparison of multiple samples (Figure 8). As an example we reanalyzed the procyclic *T. brucei *SCC1-mutAB cell line where disruption of nucleus morphology makes analysis of nucleus number less relevant (Figure 8). We have shown these cells can, following failure of mitosis, complete cytokinesis and re-enter S phase, reaching greater than four times normal nuclear DNA content.

We extended this quantitative analysis to build an automated tool capable of rapidly extracting data on kinetoplast and nucleus number, shape, position within the cell and DNA content along with cell length and width measurement from micrographs. Data collected by this method from procyclic *T. brucei *are consistent with both manual measurements and previously published data. Furthermore, we were able to quantify of growth in cell length and kinetoplast repositioning through the cell cycle (Figure 7). This type of analysis is only possible with a data set such as ours where correlation of multiple morphological measurements can be made without relying on classifications based on kinetoplast and nucleus number.

Use of an automated analysis tool, such as the one described here, accelerates analysis and reduces the risk of bias. It also ensures data collection is perfectly replicable, that there is a permanent record of all the data collected for every cell and keeps the possibility of archiving, future analysis and manual curation of data open. This data can then be used in many ways; for example it may simply be directly analyzed, or may be used to guide more extensive manual analysis of abnormal cell classes; the data from any cell may be crossreferenced back to the original micrographs. Our tool for automated image analysis therefore has potential for supporting both small-scale work and high-throughput analysis of large numbers of samples such as RNAi library [13] and drug candidate [14] screening. We have made the software tools we developed to perform these automated analyses freely available for others to use and develop further at http://users.ox.ac.uk/~path0493/htiaot.html.

Our tools for automated analysis of cell morphology did, however, have some limitations. Analysis of morphological features (length, width and kinetoplast and nucleus location) was limited to cells that did not have a branched skeleton. This would prevent partially out-of-focus cells and some mutant morphologies from being analyzed. Our approach to morphology analysis could be extended, by modifying the algorithm for analysis of the medial axis transform to take into account multiple skeleton branches, to analyze these cells. Other issues with accurate analysis of cells primarily arose from technical issues with sample preparation (debris and multiple cells lying in contact with each other) and image capture (out-of-focus cells). Potential improvements that could be made to reduce the impact of imperfections in the samples are focus stacking, to reduce the impact of out of focus cells, and taking into account image texture, from the phase contrast image, to differentiate debris and thresholding artifacts from cells.

There is clear potential to expand the capabilities of our DNA staining and image analysis tools and adapt them for related applications. Firstly the automated morphology analysis tools we have developed could be applied to other trypanosomatid organelles. The approach for measurement of kinetoplast and nucleus location could be used to measure the location of any fluorescently labeled structure in the cell. Similarly the MAT-based morphology analysis could be used to analyze morphology of the flagellum, if it were fluorescently labeled, giving automated measurement of flagellum length, width and curvature. Furthermore, the MAT cell shape analysis could be adapted to measure the distribution of a diffuse fluorescent stain through the cell. Secondly, the principle of using two DNA stains and color deconvolution for separate analysis of kinetoplast and nuclear DNA could be adapted to any system capable of analyzing fluorescence. For example, kinetoplast and nucleus DNA could be analyzed separately by flow cytometry using double DNA staining and zoids (kinetoplast DNA only) and dyskinetoplastic cells (nuclear DNA only) to determine the reference values for signal deconvolution. Finally, the approach of using two DNA stains and color deconvolution can be applied to any biological sample with regions of different DNA sequence bias. Examples include AT/GC rich banding in condensed chromosomes and AT or GC rich regions of the interphase eukaryotic nucleus arising from the underlying nuclear organization. The source code for our color deconvolution and morphology analysis tools are freely available for others to modify to enable these kinds of analyses.

## Conclusions

Our application of double DNA staining and color deconvolution of the resulting fluorescence micrographs provides new tools for the analysis of trypanosomatid morphology and DNA both manually and automatically. This technique provides three key advantages over pre-existing fluorescence microscopy methods. Firstly, it provides a method for the unambiguous identification of kinetoplast and nuclear DNA in micrographs based only on the sequence bias of the DNA present in the organelle. Secondly, it simplifies quantitation of kinetoplast and nuclear DNA from micrographs by both manual and automatic methods, including for aberrant morphologies. Finally, the separation of kinetoplasts and nuclei to separate images greatly simplifies automated analysis of trypanosomatid cell morphology and we have developed automated morphology analysis tools to take advantage of this. Furthermore the reagents required for this technique are low in cost and the image analysis is not particularly computationally intensive making it an accessible technique for the typical research environment.

## Methods

### Cell lines and culture

Promastigote form *L. mexicana *(WHO strain MNYC/BZ/62/M379) were grown in M199 medium (Sigma-Aldrich, Gillingham, UK) (10% fetal calf serum (FCS), pH 7.4, 28°C). Procyclic form *Trypanosoma brucei brucei *(Lister 427) were grown in SDM-79 medium [45]. Choanomastigote form *Crithidia fasciculata *were grown in Brain and Heart Infusion medium (Sigma-Aldrich) (5% FCS, pH 7.4, 28°C). All species were maintained at culture densities between 1 × 10^5 ^and 1 × 10^7 ^cells/ml by repeated subculture, culture density was measured with a CASY model TT cell counter (Roche Diagnostics, Burgess Hill, UK). The non-cleavable SCC1 mutant allele (SCC1-mutAB; [39]) was expressed in procyclic form *T. brucei *from pDEX-377 [46] by addition of 1 µg/ml doxycycline to the culture for 48 h.

### DNA staining and microscopy

Microscopy samples were prepared as follows (more detailed instructions are available for download from http://users.ox.ac.uk/~path0493/htiaot.html). *T. brucei*, *L*. *mexicana *and *C. fasciculata *were harvested from culture by centrifugation, washed with phosphate-buffered saline (PBS) and fixed with 2% paraformaldehyde in PBS. Cells were permeabilized with -20°C methanol and rehydrated with PBS. Many base pair intercalating stains also label RNA, therefore samples were incubated with 50 μg/ml RNaseA at room temperature for 1 h prior to staining. For analysis of a single sample (method 1) cells were stained with a combination of 1 μg/ml DAPI or bisbenzimide (Hoechst) 33342 and 40 μg/ml PI or 1:10,000 SYBR green (Sigma-Aldrich, S9430) for 2 min, washed with PBS and mounted in glycerol with 1% 1,4-diazabicyclo[2.2.2]octane and 10% 50 mM sodium phosphate, pH 8.0. Images were captured with an HCX PL APO 40 × NA 1.25 oil immersion lens (Leica Microsystems, Milton Keynes, UK) on a DM5500 B epifluorescence microscope (Leica Microsystems) with an Orca cooled CCD camera (Hamamatsu Photonics, Welwyn Garden City, UK). Images were only captured from regions of the sample not pre-exposed to the fluorescence light source; this avoids photobleaching and therefore allows accurate quantification of DNA from the fluorescent signal.

For comparative analysis of multiple samples (method 2) slides were stained by immersion in 40 ng/ml DAPI and 200 ng/ml PI in PBS for 2 min then mounted in the staining solution. This method aims to minimize variation, that is, give maximum precision, by keeping the DNA within the sample in equilibrium with the same concentration of the fluorescent DNA stains at all times. This is at the cost of photostability of the fluorescent stains as no photoprotective agent is present.

### Automated micrograph analysis

Automated image analysis was performed in ImageJ [28]. Our tools for automated correction chromatic aberration, color deconvolution and automated micrograph analysis were written in the ImageJ macro language and are described below. The automated analysis tools and usage instructions are available for download from http://users.ox.ac.uk/~path0493/htiaot.html.

### Correction of chromatic aberration

Measurement and correction of chromatic aberration uses a simplified version of the method described by [38]. Our tool used a maxima finding algorithm to find bright objects (that is, kinetoplasts and nuclei) in the MGB stain and BPI images. Each point in the MGB image is partnered with its nearest neighbor in the BPI image; this is likely to be the same kinetoplast or nucleus but offset due to chromatic aberration. Once placed into pairs the shifts in the × (∂*_x_*) and y (∂*_y_*) direction were calculated. As linear scaling around a point (that is, a difference in magnification) is sufficient to correct for lateral chromatic aberration [38] then the horizontal difference in positions of a point as seen in the MGB and BPI images, *x_MGB _*- *x_BPI _*= ∂*_x_*, will have a linear relationship with the horizontal position of the point in the MGB image, *x_MGB_*. The same will also be true for the vertical direction. The values for the scale factor and center around which to perform the scaling were therefore given by the result of linear least squares regression between the location of a particle (*x_MGB _*or *y_MGB_*) and the distance it shifts by when viewed at the second fluorescence wavelength (∂*_x _*or ∂*_y_*). Consider linear regression of the horizontal component of the aberration with horizontal position in the image. This gives a regression coefficient, *m_x_*, and vertical axis intercept, *c_x_*:

$$\partial_{x}=m_{x}x_{MGB}+c_{x}$$

The regression coefficient of the fitted line gives the scale factor in the × direction, *m_x _*+ 1, and the center around which to perform the scaling is given by -*m_x_*/*c_x_*. These factors were calculated by the same method for the vertical direction and give the scale transformation factors required to correct for chromatic aberration. The aberration was then corrected by the scaling up of the BPI image with bicubic interpolation.

### Color deconvolution

Mathematically two-dimensional color deconvolution is a change in basis in two dimensions and acts to reassign signal from two images according to two reference vectors. Our tool applies the following equation to every pixel in the input images:

$${\vec{p}}^{\prime }=M^{-1}\vec{p}$$

Where $\vec{p}$ is a vector representing the value of the current pixel in the minor groove binding DNA stain (*p_MGB_*) and base pair intercalating DNA stain (*p_BPI_*) images, $\vec{p}=\left({}_{p_{BPI}}^{p_{MGB}}\right)$ and ${\vec{p}}^{\prime }$ is a vector representing the resulting pixel values in the nucleus (*p_NUC_*) and kinetoplast (*p_KIN_*) images produced, $\vec{p^{\prime }}=\left({}_{p_{KIN}}^{p_{NUC}}\right)$. The transformation matrix is made up of reference values that describe the two-dimensional color of kinetoplasts and nuclei as seen in the MGB and BPI images:

$$M=\left(\vec{k},\vec{n}\right)=\left(\begin{array}{cc} k_{BPI} & n_{BPI}\\ k_{MGB} & n_{MGB} \end{array}\right).$$

In order to calculate the reference values of *k_BPI_*, *n_BPI_*, *k_MGB _*and *n_MGB _*our tool used a maxima finding algorithm to find bright points, that is, kinetoplasts and nuclei, present in either of the two DNA fluorescence images and measured the intensity of those points in both the MGB and BPI fluorescence images. For every point the log_2 _MGB to BPI intensity ratio was calculated and *k-*means clustering was used to assign each point to either the high log_2 _ratio or low log_2 _ratio category corresponding to kinetoplasts and nuclei, respectively. We used the log_2 _intensity ratio for classifying kinetoplasts and nuclei as it is only sensitive to the sequence bias of the organelles and is not influenced by the total DNA quantity present. The average signal intensity in the MGB and BPI images for both the kinetoplast and nucleus cluster gives the values of *k_BPI_*, *n_BPI_*, *k_MGB _*and *n_MGB_*.

### Other methods for DNA analysis

Manual image analysis was performed in ImageJ [28]. Measurement of DNA content of kinetoplasts and nuclei was made from the DAPI fluorescence image; kinetoplasts and nuclei were manually outlined and the sum pixel intensity in the outline region was measured. Flow cytometry was performed using PI for the DNA stain as described in [47].

## Competing interests

The authors declare that they have no competing interests.

## Authors' contributions

RJW conceived the DNA staining approach and wrote the automated analysis tools. KG and EG designed the validation experiments which RJW performed. All authors contributed to analysis of the data. RJW wrote the paper and all authors contributed to revising it. All authors read and approved the final manuscript.

## Supplementary Material

Additional file 1 **Figure S1**. Double labeling of kinetoplasts and nuclei with minor groove binding and base pair intercalating DNA stains. Click here for file

Additional file 2 **Figure S2**. Correcting chromatic aberration is important for accurate color deconvolution. Click here for file

Additional file 3 **Figure S3**. Measurement and correction of chromatic aberration in fluorescence images of kinetoplastid DNA. Click here for file

Additional file 4 **Figure S4**. Double staining of DNA has low variation across samples prepared in parallel. Click here for file

Additional file 5 **Figure S5**. Screenshots of the ImageJ analysis macros in use. Click here for file
