# Supplementary material for: Detailed interrogation of trypanosome cell biology via differential organelle staining and automated image analysis
Source: BMC Biol. 2012 Jan 3;10:1. doi: 10.1186/1741-7007-10-1 (PMC3398262; doi:10.1186/1741-7007-10-1)
Supplement: Additional file 1 — Figure S1. Double labeling of kinetoplasts and nuclei with minor groove binding and base pair intercalating DNA stains. [file 1741-7007-10-1-S1.PDF]

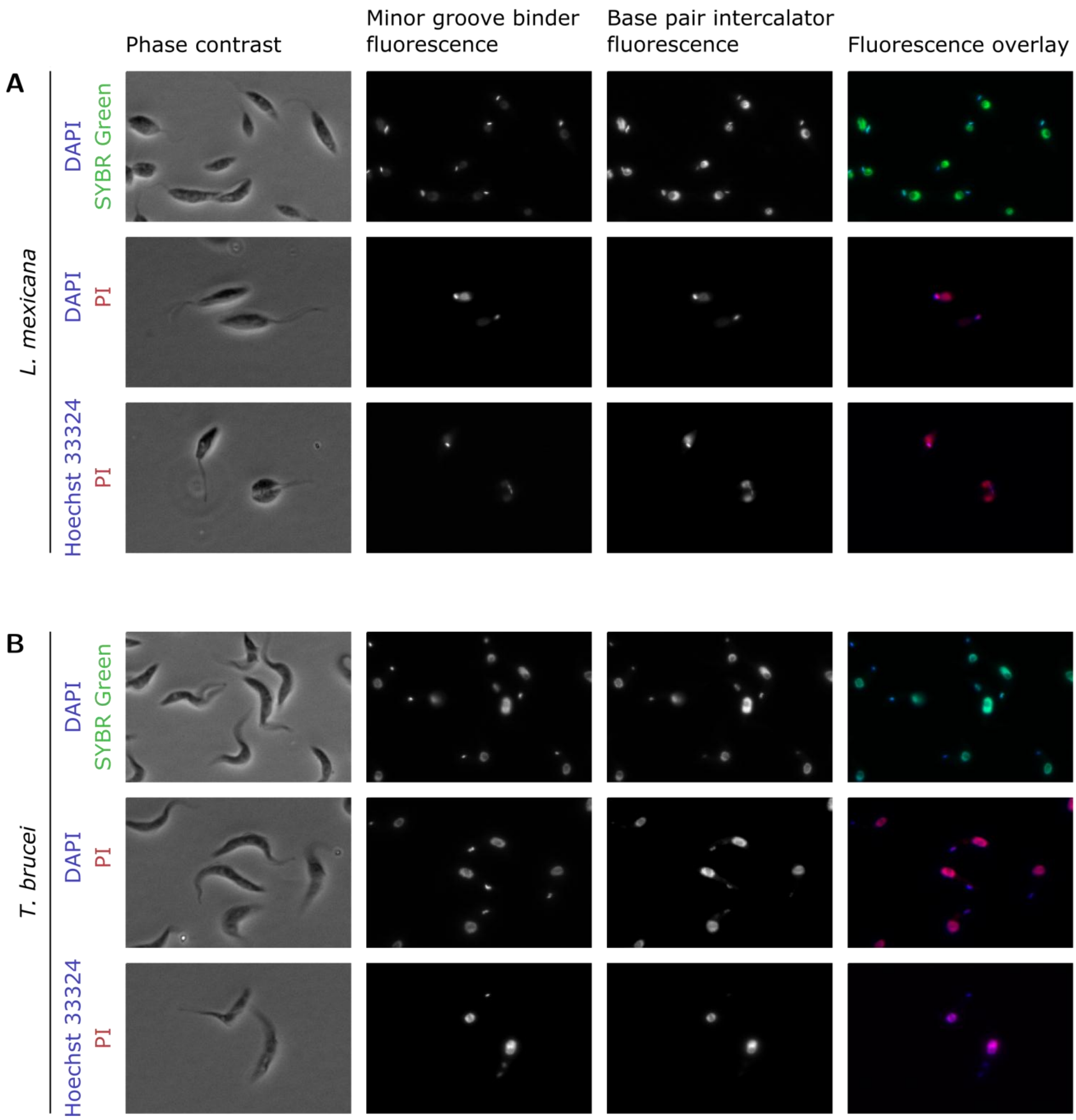


Continued on page 2

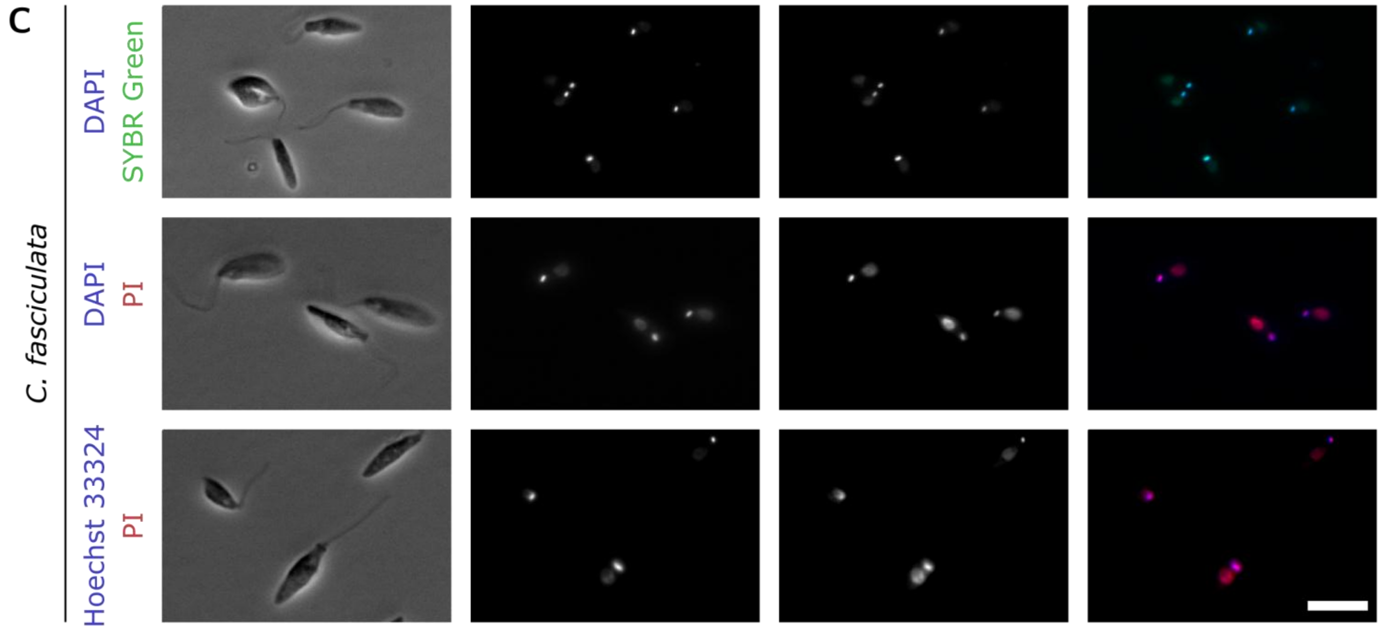


**Figure S1. Double labelling of kinetoplasts and nuclei with minor groove binding and base pair intercalating DNA stains.** This figure extends figure 1. Fluorescence micrographs of three kinetoplastid species labelled with different combinations of fluorescent DNA stain. Minor groove binding DNA stains, DAPI and Hoechst 33324, have a binding bias towards A-T rich DNA as found in the kinetoplast. Base pair intercalating DNA stains, PI and SYBR green, do not have this bias. In the fluorescence overlay of DAPI or Hoechst 33324 (blue) and PI or SYBR green (red or green respectively) this bias can be seen causing the kinetoplasts to appear more blue. This effect occurs for all stain combinations and species. Scale bar represents 10 µm.
