# Supplementary material for: Detailed interrogation of trypanosome cell biology via differential organelle staining and automated image analysis
Source: BMC Biol. 2012 Jan 3;10:1. doi: 10.1186/1741-7007-10-1 (PMC3398262; doi:10.1186/1741-7007-10-1)
Supplement: Additional file 2 — Figure S2. Correcting chromatic aberration is important for accurate color deconvolution. [file 1741-7007-10-1-S2.PDF]

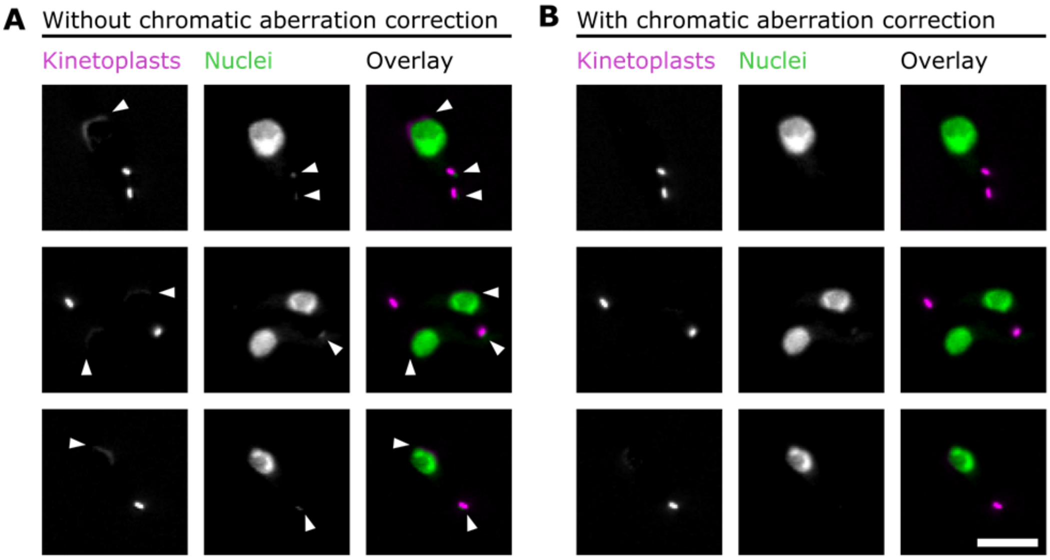

Figure S2. Correcting chromatic aberration is important for accurate colour deconvolution. A. Examples of procyclic *T. brucei* labelled with DAPI and PI following colour deconvolution without correction for chromatic aberration. The slight misalignment of the DAPI and PI images resulted in fringes around the kinetoplasts and nuclei following colour deconvolution (arrowheads). **B.** The same three example cells following colour deconvolution with correction for chromatic aberration. This removed the fringes around the kinetoplasts and nuclei. Scale bar represents 5 µm.
