# Supplementary material for: Detailed interrogation of trypanosome cell biology via differential organelle staining and automated image analysis
Source: BMC Biol. 2012 Jan 3;10:1. doi: 10.1186/1741-7007-10-1 (PMC3398262; doi:10.1186/1741-7007-10-1)
Supplement: Additional file 3 — Figure S3. Measurement and correction of chromatic aberration in fluorescence images of kinetoplastid DNA. [file 1741-7007-10-1-S3.PDF]

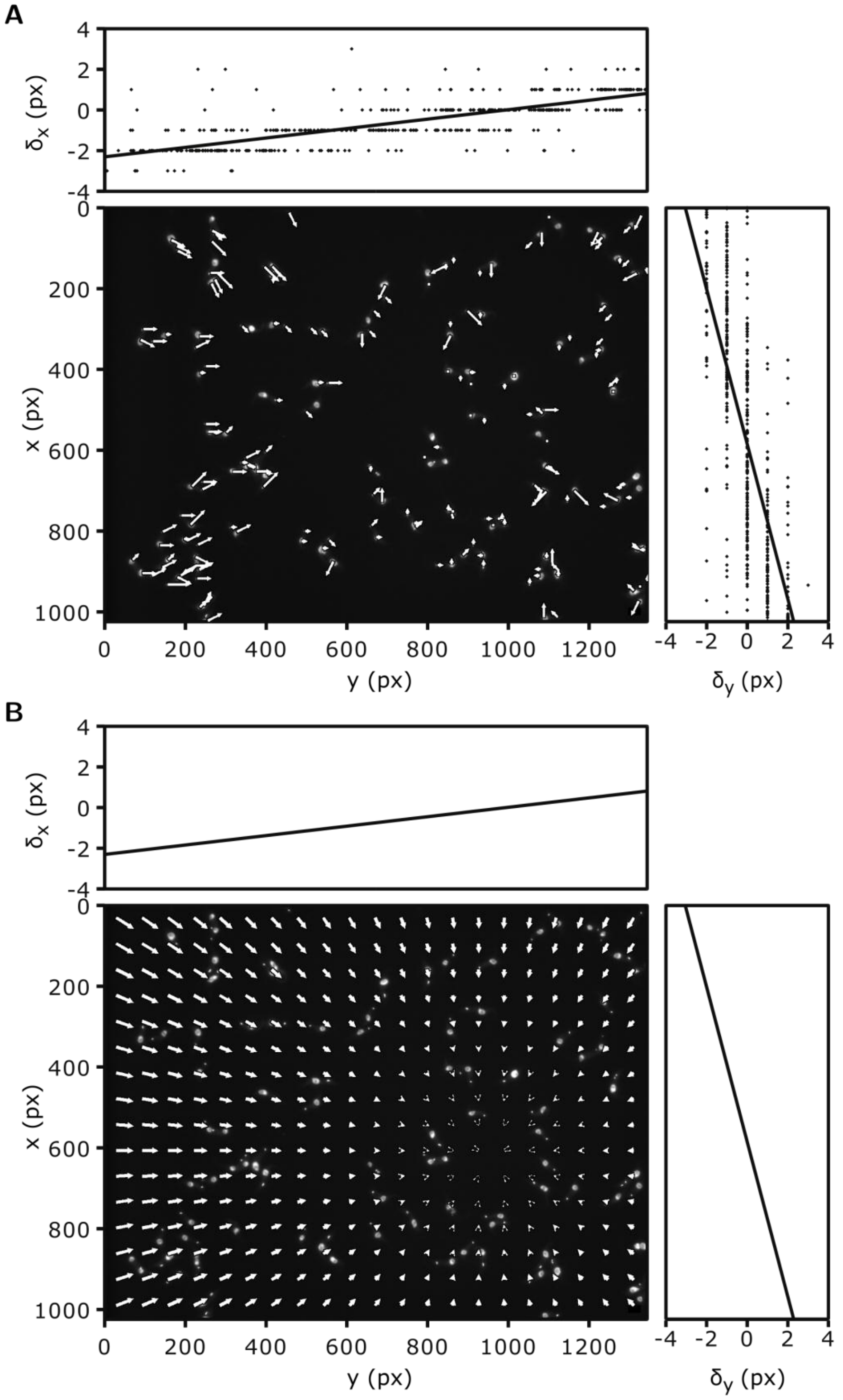

Figure S3. Measurement and correction of chromatic aberration in fluorescence images of kinetoplastid DNA. Chromatic aberration is measured by finding bright particles (kinetoplasts and nuclei) in the two fluorescent DNA stain images then measuring the shift in location between the channels. **A.** Example data from DAPI and PI stained procyclic *T. brucei*. The central image plot shows a DAPI fluorescence image; arrows indicate the direction and extent of the shift in a particle's location between the DAPI and PI fluorescence images, exaggerated by a factor of 20. The two peripheral scatter plots show the combined data from three fields of view and demonstrate particle location in the DAPI image (x_{MGB} and y_{MGB}) correlates with displacement in particle position between the DAPI and PI images (δ_x and δ_y). The least squares linear regression lines are shown. **B.** Taken together the regression lines in **A** indicate a scaling of the DAPI image by a factor of 1.0023 in the horizontal direction and 1.0024 in the vertical direction around the point (995.9, 622.8) will correct for chromatic aberration between DAPI and PI fluorescence images in these images. This transformation is shown in the central plot as an array of arrows indicating the direction of shift at that point in the image, exaggerated by a factor of 20.
