# Supplementary material for: Detailed interrogation of trypanosome cell biology via differential organelle staining and automated image analysis
Source: BMC Biol. 2012 Jan 3;10:1. doi: 10.1186/1741-7007-10-1 (PMC3398262; doi:10.1186/1741-7007-10-1)
Supplement: Additional file 4 — Figure S4. Double staining of DNA has low variation across samples prepared in parallel. [file 1741-7007-10-1-S4.PDF]

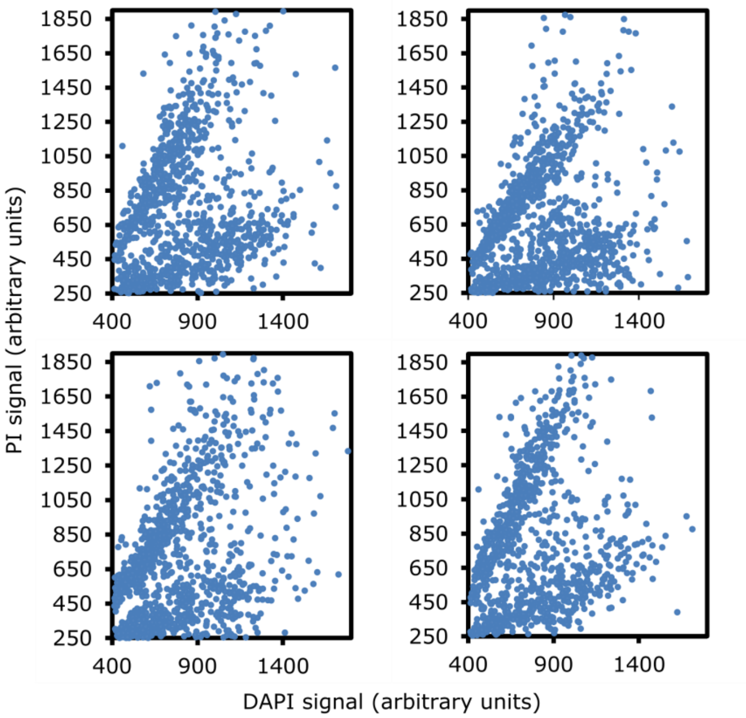

Figure S4. Double staining of DNA has low variation across samples prepared in parallel. Double staining of DNA can be optimised to have low variability. Four sets of 30 images of DAPI and PI stained procyclic *T. brucei* were captured from parallel samples on a single slide with the same exposure times. The values of 1300 bright points (kinetoplasts and nuclei) in the DAPI and PI images are shown for each image set. There is little variation in staining across the slide.
