# Supplementary material for: Detailed interrogation of trypanosome cell biology via differential organelle staining and automated image analysis
Source: BMC Biol. 2012 Jan 3;10:1. doi: 10.1186/1741-7007-10-1 (PMC3398262; doi:10.1186/1741-7007-10-1)
Supplement: Additional file 5 — Figure S5. Screenshots of the ImageJ analysis macros in use. [file 1741-7007-10-1-S5.PDF]

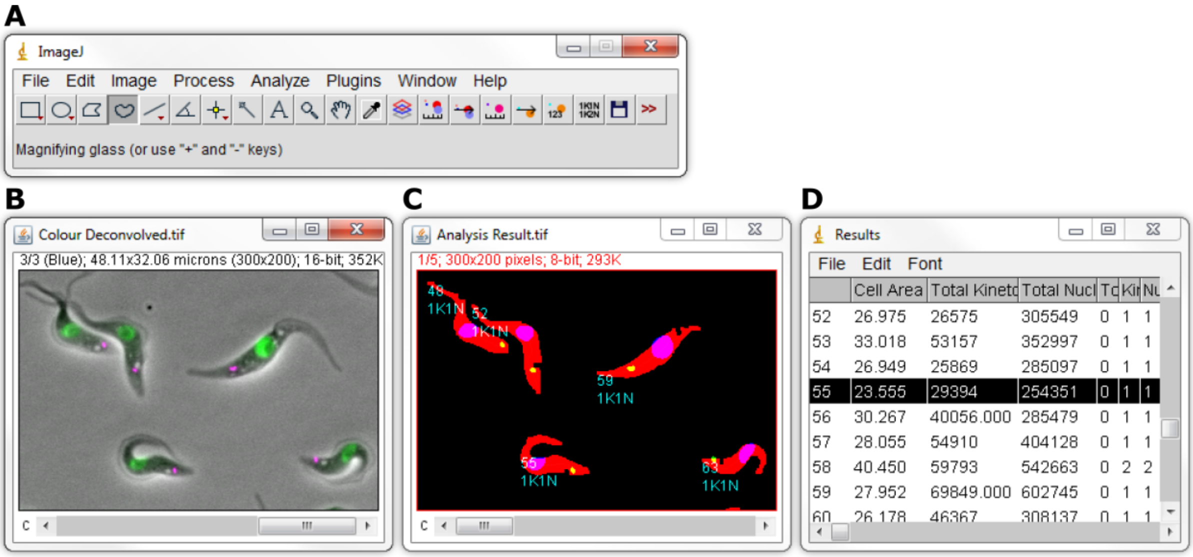

Figure S5. Screenshots of the ImageJ analysis macros in use. A. The main window of ImageJ with the analysis macros installed. The eight icons on the right of the toolbar are the analysis tools, left to right: image setup, measure chromatic aberration, correct chromatic aberration, measure K/N signal, colour deconvolution, cell analysis, K/N count summary and save analysis. **B.** A section of an example colour deconvolved image displayed as an overlay of phase contrast, kinetoplasts (cyan) and nuclei (green). This is the type of image generated by the colour deconvolution tools and is used as the input for the automatic analysis of cell morphology and DNA content. **C.** The image generated by analysis of the input image in **B**. The automatically identified cells are shown in red, kinetoplasts in yellow and nuclei in magenta. The cell ID and the automatically determined kinetoplast and nucleus number are shown in cyan text. **D.** Part of the data table generated during analysis of the input image in **B**. Each row corresponds to the data from a single cell. The data from cell ID 55 (bottom centre in **B** and **C**) is highlighted.
